# Early Post-Operative Intervention of Whole-Body Vibration in Patients After Total Knee Arthroplasty: A Pilot Study

**DOI:** 10.3390/jcm8111902

**Published:** 2019-11-07

**Authors:** Yu-Hsuan Hsiao, Song-Hsiung Chien, Hung-Pin Tu, Jimmy Chun-Ming Fu, Shih-Ting Tsai, Ying-Shan Chen, Yi-Jen Chen, Chia-Hsin Chen

**Affiliations:** 1 Department of Physical Medicine and Rehabilitation, Kaohsiung Medical University Hospital, Kaohsiung 807, Taiwan; wannahappy39@gmail.com (Y.-H.H.); jimmy90295@hotmail.com (J.C.-M.F.); alisontsai0329@gmail.com (S.-T.T.); chordata.chen@gmail.com (Y.-S.C.); 2 Department of Orthopaedics, Kaohsiung Medical University Hospital, Kaohsiung 807, Taiwan; schien@kmu.edu.tw; 3 Department of Public Health and Environmental Medicine, School of Medicine, College of Medicine, Kaohsiung Medical University, Kaohsiung 807, Taiwan; 4 Department of Physical Medicine and Rehabilitation, School of Medicine, College of Medicine, Kaohsiung Medical University, Kaohsiung 807, Taiwan; 5 Graduate Institute of Clinical Medicine, College of Medicine, Kaohsiung Medical University, Kaohsiung 807, Taiwan; 6 Department of Physical Medicine and Rehabilitation, Kaohsiung Municipal Ta-Tung Hospital, Kaohsiung 801, Taiwan; 7 Orthopaedic Research Center, Kaohsiung Medical University, Kaohsiung 807, Taiwan

**Keywords:** whole-body vibration, total knee arthroplasty, strength, swelling

## Abstract

(1) Background: Knee osteoarthritis causes pain, weakness, muscle atrophy, and disability. The application of whole-body vibration in patients with knee osteoarthritis can improve strength, balance, and functional activities. The purpose of the study is to evaluate the effects of early whole-body vibration intervention in patients after total knee arthroplasty. (2) Method: A single-blinded randomized control trial. Fifty-two patients with knee osteoarthritis post total knee replacement from a medical center in southern Taiwan were randomly assigned to either a whole-body vibration group or control group. Main outcome measures included pain severity, leg circumference, knee range of motion, knee extensor strength, a five-times sit to stand test, and a timed up and go test. (3) Results: Immediately post treatment, the patients in the vibration group showed a significant increase in knee extensor strength and improvement in calf swelling compared to the control group. A trend toward decrease in pain severity and improvement in functional performance were observed in both groups without a significant difference between the groups. There was no significant difference in knee range of motion (ROM) and functional performance between the groups. (4) Conclusions: The whole-body vibration intervention in patients early post total knee arthroplasty showed significant immediate effect in increasing knee extensor strength and decreasing calf swelling.

## 1. Introduction

Knee osteoarthritis (OA), a degenerative joint disease, is a leading cause of disability in developed countries [1]. The prevalence of knee OA increases with age, especially in women. In Taiwan, about 37% of people over the age of 50 have OA. Symptomatic knee OA results in arthralgia, joint stiffness, and limited joint range of motion (ROM), leading to muscle weakness in the lower extremity, disability, and limitation in activities of daily living in the elderly population [2]. Clinical managements for knee OA include pharmacological treatments with oral painkillers, intra-articular corticosteroids, intra-articular hyaluronic acid injection and non-pharmacological managements with transcutaneous electrical nerve stimulation, aerobic exercise, resistance exercises of lower limbs, proprioception training, and weight control [3,4]. Advanced knee OA treated with total knee arthroplasty (TKA) could effectively reduce pain, help patients return to activities of daily living, restore mechanical alignment, and preserve the joint lines [5,6].

Although there is a high success rate with TKA, patients after TKA usually do not have restored normal knee function and still experienced functional impairment [7,8], and early quadricep strength loss of 62% was seen one month post-operatively, primarily related to failure of voluntary muscle activation followed by muscle atrophy and knee pain [9]. Post-TKA knee swelling is one of the factors causing decreased knee extensor strength and functional performance after operation [10,11]. Exercise regimens that emphasize quadricep muscle contraction and clinical managements that facilitate muscle activation are necessary to improve quadricep strength and functional performance in patients post-TKA [12,13]. Conventional rehabilitation with muscle strengthening, continuous passive motion (CPM), therapeutic exercise, and gait training after TKA can improve pain, ROM, hospital stay length, and functional activity [14]. However, patients usually refrain from exercise and active knee contraction due to post-operative pain and leg swelling. Strategies to reduce post-operative pain and swelling are important to early encountering of exercise training in patients after TKA.

Low frequency, low amplitude mechanical stimuli using a vibrating platform could stimulate the muscle spindles via tonic vibration reflex and send nerve impulses to increase muscle contraction [15]. Whole body vibration (WBV) exercise has been suggested as an alternative treatment to reduce pain and improve muscle strength, balance, and flexibility in sedentary and older people [16,17,18,19]. In knee OA, the effects of WBV in improving knee pain, quadricep strength, and functional performance remain inconclusive [20,21], however, recent randomized controlled trials provided evidence of additive effects of WBV in increasing knee extensor strength and improving physical functions [22,23,24]. Besides this, both WBV strengthening and conventional progressive resistance exercise can improve knee extensor strength and functional activity in TKA patients during subacute phase [25]. However, the effect of early WBV intervention on patients after TKA remains unknown.

Our purpose of the study is to determine if the early intervention of low frequency, low amplitude WBV along with conventional physical therapy could enhance the rehabilitation effect on patients in the early stages of post-TKA.

## 2. Experimental Section

### 2.1. Study Population

The study was approved by the Institutional Review Board of our hospital (KMUH-IRB-F(II)-20170135) and registered in ClinicalTrials.Gov ID-NCT04107350. The study was carried out from March 2018 to February 2019. The participants were enrolled from the orthopedic ward. The inclusion criteria were as follows: Knee OA status post TKA, age between 55–90 years old, and the ability to walk with or without assistant devices before operation. Those with the following conditions were excluded: Uncontrolled blood pressure, known neoplasm, cardiac pacemaker, cardiovascular disease, respiratory disease, cerebrovascular disorder, or impaired verbal expression or comprehension. The study protocol was well explained to the participants and informed consent was given by the participants. The participants enrolled were randomly allocated to one of two parallel groups, the WBV group or the control group, in a 1:1 ratio.

### 2.2. Design of Post-Operative Exercise Interventions

The study was designed as a single-blinded, randomized controlled trial. The patients were treated by physical therapists and evaluated by another physician who was blinded to their allocation. All enrolled participants with knee OA status post-TKA were randomly allocated to either the WBV group or the control group on the post-operative day 1. Both groups received conventional physical therapy, including muscle strengthening, passive ROM exercise with CPM machine, active ROM exercise, and activities of daily living training, including transfer, walking, and going up/down stairs after the operation. All participants received analgesics and/or anti-inflammatory drugs to reduce pain during the early post-operative period.

**Figure 1:**
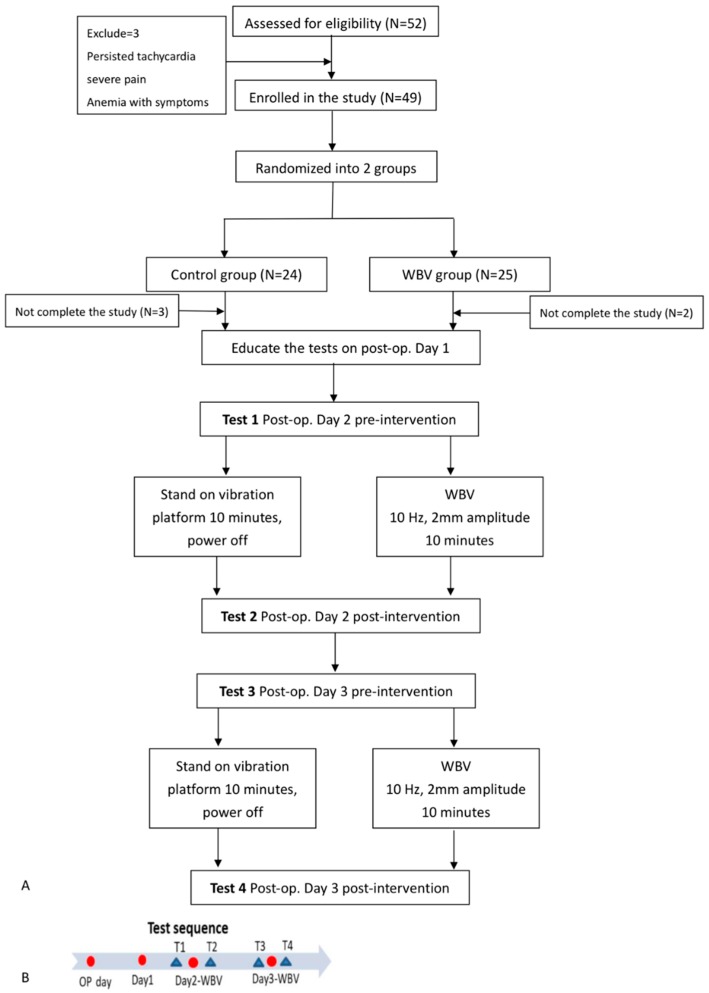
(**A**) Flowchart of the study. WBV, whole body vibration, OP, operation. (**B**) Test sequence. OP, operation; T1, pre-intervention assessment on post-operative day 2; T2, post-intervention assessment on post-operative day 2; T3, pre-intervention assessment on post-operative day 3; T4, post-intervention assessment on post-operative day 3.

In the WBV group, participants received one session per day, 2 sets per session of five-minute WBV at low frequency (8–10 Hz) and low amplitude (2 mm) in a vertical direction (BW-750, Bodygreen, Taiwan), with a three-minute rest interval during the post-operative day 2 and day 3. During the intervention, participants were positioned on the vibration platform in a standing position with hands on the walker to prevent falls. During the vibration, participants were corrected to maintain an upright position and bear weight evenly on bilateral feet. In the control group, participants received the same procedures, with the vibration machine off. The flowchart of the study is shown in Figure 1A.

### 2.3. Outcome Measures

The outcome measures included pain severity assessed with numerical rating scale (NRS), active range of motion (AROM) and passive range of motion (PROM) of the operated knee, leg swelling evaluation with thigh and calf circumference, operated knee extensor strength, and functional performance assessment using a five-time sit to stand test (5TSTS) and timed up and go test (TUG).

During the post-operative day 1, participants were educated on the intervention and assessment procedures. During the whole intervention, participants received a total of four assessments, including pre-intervention assessment on post-operative day 2 (T1), immediate post-intervention assessment on post-operative day 2 (T2), pre-intervention assessment on post-operative day 3 (T3), and immediate post-intervention assessment on post-operative day 3 (T4). A schematic summary of the study protocol is shown in Figure 1B.

#### 2.3.1. Pain Scale (0–10)

The pain severity was evaluated using NRS rating from 0 to 10, with 0 representing no pain and 10 representing maximal pain. Any discomfort during the WBV was recorded.

#### 2.3.2. Knee Joint Range of Motion

The knee flexion AROM and PROM of the operated knee were measured using a goniometer in the supine position. The center of the goniometer was placed on the lateral femoral condyle of the knee joint. The lateral malleolus was used as a distal reference marker, and the greater trochanter was used as a proximal reference point [26].

#### 2.3.3. Leg Circumference

To evaluate the degree of leg swelling, we measured the thigh and calf circumferences in centimeters. The fibular head (FH) was used as a landmark. The thigh circumference was measured at 15 centimeters above FH, and the calf circumference was measured at 5 centimeters below FH. All measurements were done in a supine position.

#### 2.3.4. Knee Extensor Muscle Strength

We used a MicroFET2 hand-held dynamometer (Hoggan Scientific, LLC., Salt Lake City, UT, USA) to measure the isometric knee extensor strength of the operated knee. This portable device is a discrete and reliable measurement tool to objectively measure muscle strength, compared to traditional manual muscle testing. For strength measured by the hand-held dynamometer, kilograms (kg) was considered the unit of measurement [27]. A previous study has reported increased reliability and validity of quadricep strength measurements using a fixed hand-held dynamometer in the supine position [28], and maximal voluntary contraction torque of the quadriceps muscle occurred at mid-range of knee ROM (45–90-degree flexion) [29]. In this study, maximal isometric knee extensor strength was measured with participants in the supine position and the knee joint flexed at 45 degrees, fixed on a triangle block. The tests were done three times and the average value of the isometric peak flexion force (kg) was recorded.

#### 2.3.5. Functional Performance Test with Modified Five Times Sit to Stand Test and Modified Timed Up and Go (TUG) Test

**• Modified five times sit to stand test**

We used a straight back chair with a solid seat 40 cm high. We asked the participants to stand up and sit down as quickly as possible five times, and the time spent was recorded. A previous study did a modified 30-second sit to stand test that allowed upper extremity use in older adults to screen for fall risk in long-term care [30]. Concerning the risk of fall and pain in the early post-operative status, we modified the test to ask participants to hold their hands on the walker while performing the test. Participants were allowed one practice trial before the actual measurement.

**• Modified TUG test**

The TUG test has been used as an outcome measurement following therapy or surgery and as a predictor of falls and function. It could assess strength, agility, and dynamic balance during multiple activities [31]. Participants were asked to stand up from a standard chair with seat height of 40 cm, walk a distance of 3 meters, turn around, walk back to the chair, and sit down. The time (in seconds) spent was recorded. The participants were allowed to use a walker as an assisted device during the test to prevent falls.

### 2.4. Statistics

The continuous and categorical variables were analyzed by *t* test and the Fisher exact test, respectively, for comparisons between WBV and control groups. The primary efficacy analysis according to the statistical analysis plan included all randomized participants (intention-to-treat principle) and used last observation carried forward imputation. The generalized estimating equation model (GEE) was used for repeated measures for the change in mean differences between WBV and control groups. We used SAS (version 9.4; SAS Institute, Cary, NC, USA) for all analyses. A value of *p* < 0.05 was regarded as statistically significant. We calculated the sample size of 19 patients per group with the assumption of a mean quadricep strength between the two groups of 1.20 units (standard deviation (SD) 1.25), which provided 80% power to detect such a difference with a two-sample *t* test with a two-sided type I error of 0.05. We therefore aimed to recruit 48 participants (approximately 24 per group, with assumed dropout rate of 20%) to achieve roughly 19 patients per treatment group.

## 3. Results

### 3.1. Characteristics of the Participants 

Fifty-two patients were consecutively recruited and three were excluded due to unstable vital signs, severe pain, and dizziness related to anemia. Finally, 49 participants were enrolled, where 24 participants were allocated to the control group, and 25 participants were allocated to the WBV group. In the WBV group, two participants could not complete the isometric knee extensor strength testing on post-operative day 2 and day 3 due to pain provoked and wound oozing; in the control group, 3 participants failed to complete the tests due to anemia-related dizziness, anemia-related dyspnea and post-operative wound pain. All other participants completed the interventions and all pre-intervention and post-intervention tests. The missing data were filled with the statistic method of GEE and last observation carried forward imputation. The basic demographics and baseline data of both groups are listed in Table 1. There were no significant differences between the groups in the baseline characteristics.

### 3.2. Effect of Whole Body Vibration (WBV) on All Outcome Measures During Serial Testing

Table 2 shows the difference between the groups in all outcome measures during the four testing periods. A trend toward decrease in pain severity and increase in both AROM and PROM angles were observed in both groups, while significantly better AROM and PROM in the post-intervention assessments (T2 and T4) were noted. Increase in knee extensor strength with time was also observed in the WBV group, with more significant differences between the groups observed in T4 (*p* = 0.010). As for the leg circumference, a trend of increase in calf circumference was observed in the control group, and significantly greater calf circumference was observed in the control group than in the WBV group on post-operative day 3 (T3 and T4). The time spent in five-times sit to stand and TUG test also decreased with time, and there was no significant difference between the groups in each assessment time point.

### 3.3. Effect of Whole Body Vibration (WBV) on Pain Reduction and Lower Leg Circumference Change

Table 3 showed the changes in mean difference between baseline and follow-up assessment of all outcome measures. Both the WBV and control groups showed a slight reduction in NRS without significant difference (*p* = 0.294). However, a significant difference between the groups in the change of calf circumference from the baseline was observed; the mean change of calf circumference in the WBV group was 0.33 cm less, but 0.41 cm greater in the control group (*p* = 0.045). The thigh circumference was 0.14 cm less in the WBV group, but 0.11 cm greater than the baseline in the control group, not reaching statistical significance (*p* = 0.550).

### 3.4. Effect of Whole Body Vibration (WBV) on Knee Extensor Strength and Knee Joint Range of Motion (ROM)

**Figure 2:**
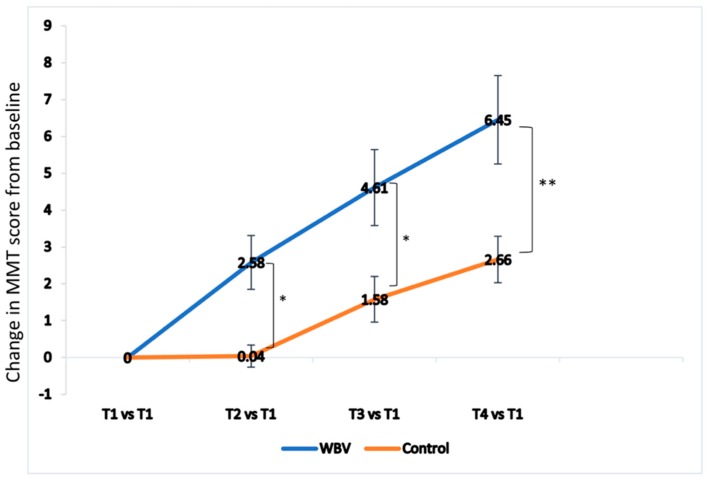
**Time course of mean change in knee extensor strength from baseline by time of treatment.** Both groups showed improvement in knee extensor strength with time. The knee extensor strength is significant higher in the whole body vibration (WBV) group. T1, pre-intervention assessment on post-operative day 2; T2, post-intervention assessment on post-operative day 2; T3, pre-intervention assessment on post-operative day 3; T4, post-intervention assessment on post-operative day 3 (T4). * *p* < 0.05, ** *p* < 0.01.

The knee extensor strength increased in both groups, and the extend of increase from baseline was more significant in the WBV group (*p* = 0.018). In the WBV group, an increase of 2.14 kg was observed, but only a 0.95 kg increase in the control group. The trend of changes in knee extensor strength compared to baseline in both groups is illustrated in Figure 2. As for the changes in knee joint flexion ROM, an increase in both AROM and PROM from the baseline was observed in both groups, without significant differences between the groups.

### 3.5. Effect of Whole Body Vibration (WBV) on Functional Performance

In both groups, the time spent on five-times sit to stand and TUG test were less when compared to the baseline, indicating improved functional performance, but not reaching significant difference between the two groups.

## 4. Discussion

In the current study, an immediate effect of early post-operative WBV intervention in increasing knee extensor strength and decreasing calf circumference was observed, and the WBV intervention was considered a safe early post-operative training method for patients after TKA.

Despite the high success rate of TKA, pre-existing quadricep weakness in knee OA and early quadricep strength loss in the post-operative status were reported. Previous studies revealed that there was up to 60% quadricep strength loss compared to the uninvolved side one month after TKA, and there is still approximately a 17% decrease in muscle activation even if post-operative rehabilitation is initiated within 24 hours after the operation. Therefore, after TKA patients usually experience persistent impairments and functional limitations [8,9]. The gait abnormality with step length asymmetry remains even at 15 weeks after surgery [32]. Poor muscle activation and muscle atrophy are two major contributors to early quadricep strength loss after TKA, and knee pain during voluntary muscle contraction plays a minor role in the reduction of muscle activation [9]. Stevens-Lapsley et al. also reported hamstring weakness and increased hamstring coactivation, compared to the non-operative leg at one month after TKA. This condition might result in hamstring tendinopathy and increase post-operation pain [33].

WBV exercises have been widely used for the benefits of pain reduction, muscle strength increase, proprioception, balance and posture control, and functional performance improvement in postmenopausal women and sedentary and older individuals [17,19,34,35,36]. A recent study investigating the effects of WBV in addition to home exercise in female patients with patellofemoral pain also reported decreased pain and improved knee extensor endurance, which may alter lower extremity kinematics and reduce patellofemoral stress [37]. In chronic knee OA, the WBV could significantly decrease knee pain, increase quadricep strength, and improve balance, compared to therapeutic exercise [34]. Several recent randomized controlled trials also suggested the additive effects of WBV in combination with resistance training in increasing knee extensor strength [22,23,24]. However, the beneficial effect of WBV on patients after TKA remains uncertain. There was only one research article investigating the effect of WBV strength training to patients after TKA. Johnson et al. showed that a four-week WBV exercise initiated in the subacute stage (3 to 6 weeks after TKA) effectively improved knee extensor strength and timed up and go scores, although not superior to those receiving traditional progressive resistance exercise, and they proposed the WBV effect to be related to less stress while doing resistance training on the vibratory platform in the elderly; however, the effect of WBV on muscle activation remains inconclusive [25]. In the current study, we investigated the immediate effect of early WBV intervention in patients after TKA. To improve tolerance of WBV during the early post-operative period, we used low frequency, low amplitude WBV (8–10Hz and 2mm amplitude) and participants were positioned in erect standing posture [38]. The study results also showed that WBV significantly increased the quadricep isometric strength with accumulated effect, compared to the control group (Figure 2).

The possible mechanisms related to the improvement in muscular performance after WBV in knee OA and post-TKA status remain unclear. Rhea et. al. reported the effectiveness of WBV in reducing delayed-onset muscle soreness in untrained individuals after strenuous exercise, and postulated that WBV may inhibit pain receptors, allowing individuals to be more tolerant to pain [39]. The effect would encourage patient to early ROM and strengthening exercise training. Another commonly proposed mechanism of WBV is the enhanced muscle activities. WBV could produce an upward thrust and cause a body movement which is in the opposite direction to gravity. Vibration stretch transmitted via Ia afferent to alpha motor neurons and activated muscle spindle receptors to increase stretch reflex and facilitate muscle contraction [40]. Therefore, WBV could increase synchronized activation of muscle spindle and motor units and improve synergist muscles and antagonist inhibition [41]. In elderly adults, low-intensity WBV effectively increased lower leg muscle activity during static standing [36]. Abbasi et al. also reported increase in electromyographic activity of lower leg muscles of patients with knee OA, in terms of better root mean square progression rate, after four weeks of WBV training [42]. Our results showed that quadriceps isometric strength increment in WBV group was significantly higher than the control group. The degree of improvement in quadriceps isometric strength in the WBV and the control groups were 45.5% and 16.7%, respectively. The acute effects observed in the current study might be associated with the enhancement of neurological mechanisms involving spinal reflex and muscle activation. Interestingly, since arthrogenic muscle inhibition is one of the key factors leading to impairments and functional limitations in patients with arthritis and following knee surgery [43], a recent study by Simao et al. proposed the contribution of neuromuscular adaptations and modulation in neuromuscular plasticity to improved muscle performance by WBV training, providing evidence of increased plasma levels of brain-derived neurotrophic factor (BDNF), one of the neurotrophins and myokines that respond to exercise [44], in elderly women with knee OA after 12 weeks of WBV training [45].

Another significant finding of the current study is the significantly decreased calf circumference in the WBV group compared to the control. Both WBV and control groups showed increased circumference on post-operative day 2, but the WBV group showed decreased thigh and calf circumference on post-operative day 3, while the control group showed progressed swelling. In the early post-operative period, regional inflammation might cause tissue swelling and disturb blood flow. Similarly, Holm et al. reported a negative correlation between post-operative knee swelling and a change in knee extension strength shortly after TKA, independent of change in knee pain [10], further proposing the critical role of arthrogenic muscle inhibition and influence of reflex inhibition of alfa-motoneurons in acute post-operative status of patients receiving TKA [43]. Lythgo et al. reported that externally applied low-frequency vibration in standing and squatting position could generate endothelial shear stress and produce nitric oxide, which can improve leg blood flow; in addition, WBV increased muscle activation and muscle contraction which might improve venous return [46]. Systematic review and meta-analysis also provided evidence of the effect of WBV in increasing peripheral blood flow and/or skin temperature of the lower extremity [47,48], and the increase in blood flow is greater at lower frequencies of WBV, which may be due to increased time between contractions that allows for greater perfusion [48]. From the current evidence, we inferred that early intervention with WBV decreased lower leg swelling in patients after TKA, potentially through increasing peripheral blood flow, enhancing muscle activation, and improving venous return stimulated by WBV.

In knee flexion ROM, both WBV and control groups had similar effects, with a slightly increased flexion angle. The CPM can effectively improve knee joint ROM, as the previous study reported. We also found a slightly better AROM increase in the WBV group, which might be related to WBV-induced increase in agonist muscle activation and antagonist inhibition. Ritzmann et al. reported that WBV could elicit more economic movement execution by simultaneous tibialis anterior contraction and soleus relaxation, leading to better functional performance [49]. Therefore, we proposed that low frequency vibration before therapeutic exercise might improve patients’ exercise performance and decrease their pain during activity.

In the functional activity evaluation of five-times sit to stand and TUG test, both groups showed less time spent as compared to the baseline assessment. We also found that the magnitude of improvement in five-times sit to stand performance was more obvious in the WBV group than in the control group (−3.72 s vs. −2.10 s), although not reaching statistical significance. The improved functional performance might be related to increased knee joint ROM, as it was reported by Holm et al. that changes in knee flexion angle were associated independently with performance in fast-speed walking [10]. Future study with a longer period of WBV intervention after TKA may be conducted to follow up its effectiveness, as a previous study reported that 8-week WBV training could improve functional performance, balance control, and flexibility in the elderly [50].

During the study periods, no significant complications were observed. Only 2 participants refused to do a knee isometric contraction test which would cause pain and mild wound oozing. There was no dizziness or discomfort reported during the intervention periods, nor hemovac dislodge or increased blood leakage amount, which indicated the tolerance of early post-operative WBV intervention in patients receiving TKA.

The limitation of the current study included a small sample size and lack of long-term follow up. Moreover, there was also a lack of pre-operative assessment, and a comparison of degree of improvement to pre-operative status is not available. A longitudinal follow up with a longer period of intervention is needed to enroll participants in the pre-operative status and perform pre-TKA assessments to assess the degree of improvement compared to baseline status and validate the clinical effects of WBV in post-TKA status.

## 5. Conclusions

Early post-operative WBV training in patients after TKA exerted immediate effects on increasing knee extensor strength and decreasing lower leg swelling. Both post-operative conventional physical therapy and WBV could reduce pain, increase knee joint flexion ROM and functional performance. WBV intervention is a modality safe and effective for patients after TKA.

## Figures and Tables

**Table 1:** Baseline characteristics of the participants in two groups.

	WBV	Control	*p*-Value
*n*	25	24	
Age, mean (SD), years	71.1 (8.0)	71.8 (5.8)	0.738
Sex, *n* (%)			
Male	4 (16.0)	5 (20.8)	
Female	21 (84.0)	19 (79.2)	0.662
Height, mean (SD), cm	154.2 (6.5)	154.0 (8.2)	0.925
Body weight, mean (SD), kg	64.2 (12.9)	67.1 (13.1)	0.445
BMI, mean (SD), kg/m^2^	27.1 (5.7)	28.2 (4.8)	0.473

SD, standard deviation; WBV, whole body vibration; BMI, body mass index.

**Table 2:** Differences between groups in serial testing of all outcome measures.

	T1			T2			T3			T4		
	WBV	Control		WBV	Control		WBV	Control		WBV	Control	
	Lsmean (SE)	Lsmean (SE)	*p*-Value	Lsmean (SE)	Lsmean (SE)	*p*-Value	Lsmean (SE)	Lsmean (SE)	*p*-Value	Lsmean (SE)	Lsmean (SE)	*p*-Value
NRS	4.12 (0.41)	3.96 (0.48)	0.793	3.68 (0.33)	3.88 (0.48)	0.733	3.40 (0.37)	2.67 (0.34)	0.147	2.88 (0.28)	2.42 (0.28)	0.239
Thigh circumference	43.56 (1.09)	44.31 (1.91)	0.725	44.55 (1.72)	45.16 (1.19)	0.767	43.72 (1.08)	44.9 (1.29)	0.476	43.36 (1.08)	44.75 (1.29)	0.400
Calf circumference	34.20 (0.75)	34.95 (1.23)	0.596	34.39 (0.69)	36.26 (0.89)	0.101	32.73 (1.16)	36.44 (0.87)	0.014	33.67 (0.71)	36.25 (0.84)	0.023
Knee extensor strength	14.21 (1.08)	13.61 (1.00)	0.726	16.79 (1.40)	13.26 (1.09)	0.051	18.83 (1.38)	14.81 (0.83)	0.017	20.67 (1.53)	15.88 (0.88)	0.010
AROM	76.20 (3.41)	70.42 (3.97)	0.265	82.4 (3.08)	72.92 (3.43)	0.044	83.60 (2.07)	78.75 (2.36)	0.124	89.80 (1.84)	81.67 (2.06)	0.024
PROM	88.80 (3.22)	81.46 (3.37)	0.117	92.4 (3.15)	83.54 (3.19)	0.053	92.20 (2.08)	88.33 (2.14)	0.194	97.60 (1.66)	92.50 (1.75)	0.039
Five-time sit to stand	39.46 (5.03)	39.13 (4.21)	0.958	36.73 (5.36)	33.77 (3.95)	0.652	33.32 (4.42)	34.19 (2.88)	0.868	28.19 (3.29)	31.98 (2.93)	0.383
TUG test	73.58 (5.75)	82.56 (10.78)	0.455	66.29 (6.43)	73.41 (10.73)	0.562	65.75 (5.72)	66.82 (5.23)	0.888	59.51 (5.12)	68.42 (8.17)	0.349

T1, pre-intervention assessment on post-operative day 2; T2, post-intervention assessment on post-operative day 2; T3, pre-intervention assessment on post-operative day 3; T4, post-intervention assessment on post-operative day 3 (T4). NRS, numerical rating scale; AROM, active range of motion; PROM, passive range of motion; TUG, timed-up-and-go; Lsmean, least squares means; SE, standard error.

**Table 3:** Changes in mean difference between baseline and follow-up assessment of all outcome measures.

	WBV Lsmean (SE)	Control Lsmean (SE)	Difference of Least Square Means (SE; 95% CI)	*p*-Value
**NRS and lower leg circumference**				
NRS baseline	4.12 (0.40)	3.96 (0.47)		
Change from baseline to 4 times	−0.40 (0.07)	−0.58 (0.16)	0.18 (0.17; −0.16 to 0.53)	0.294
Thigh circumference baseline	43.56 (1.06)	44.31 (1.87)		
Change from baseline to 4 times	−0.14 (0.17)	0.11 (0.38)	−0.25 (0.42; −1.06 to 0.57)	0.550
Calf circumference baseline	34.2 (0.73)	34.95 (1.21)		
Change from baseline to 4 times	−0.33 (0.12)	0.41 (0.34)	−0.73 (0.37; −1.45 to −0.01)	0.045 *
**Knee extensor strength and ROM**				
Knee extensor strength baseline	14.21 (1.05)	13.22 (0.98)		
Change from baseline to 4 times	2.14 (0.44)	0.95 (0.25)	1.19 (0.5; 0.20 to 2.18)	0.018 *
AROM baseline	76.20 (3.34)	70.42 (3.89)		
Change from baseline to 4 times	4.20 (0.89)	3.96 (1.11)	0.24 (1.43; −2.55 to 3.04)	0.865
PROM baseline	88.80 (3.15)	81.46 (3.30)		
Change from baseline to 4 times	2.62 (0.95)	3.79 (1.00)	−1.17 (1.38; −3.88 to 1.54)	0.397
**Functional activities**				
Five time sit to stand baseline	39.46 (4.93)	39.13 (4.12)		
Change from baseline to 4 times	−3.72 (1.08)	−2.10 (1.08)	−1.62 (1.53; −4.62 to 1.38)	0.290
TUG test baseline	73.58 (5.64)	82.56 (10.55)		
Change from baseline to 4 times	−4.27 (1.46)	−4.90 (2.05)	0.63 (2.51; −4.29 to 5.55)	0.803

NRS, numerical rating scale; ROM, range of motion; AROM, active range of motion; PROM, passive range of motion; TUG, timed-up-and-go; WBV, whole body vibration; Lsmean, least squares means; SE: Standard error; CI, confidence interval. *, *p* < 0.05.
